# A rare case of atropine-resistant bradycardia following sugammadex administration

**DOI:** 10.1186/s40981-020-00326-7

**Published:** 2020-03-02

**Authors:** Takayuki Yoshida, Chisato Sumi, Takeo Uba, Haruka Miyata, Takeshi Umegaki, Takahiko Kamibayashi

**Affiliations:** 1 Department of Anesthesiology, Kansai Medical University Hospital, 2-3-1 Shin-machi, Hirakata-city, Osaka 573-1191 Japan; 2 Department of Anesthesiology, Chibune General Hospital, 3-2-39 Fuku-machi, Nishiyodogawa-ku, Osaka-city, Osaka 555-0034 Japan; 3 Department of Anesthesiology, Baba Memorial Hospital, 4-244 Hamadera-funaocho-higashi, Nishi-ku, Sakai-city, Osaka 592-8555 Japan

**Keywords:** Sugammadex, Bradycardia, Cardiac arrest, Anaphylaxis, Coronary vasospasm, Kounis syndrome

## Abstract

**Background:**

Profound bradycardia caused by sugammadex has been reported, although its mechanism is unclear. Herein, we suggest a possible culprit for this phenomenon.

**Case presentation:**

A 50-year-old woman without comorbidity except mild obesity underwent a transabdominal hysterectomy and right salpingo-oophorectomy. After surgery, sugammadex 200 mg was intravenously administered. Approximately 4 min later, her heart rate decreased to 36 bpm accompanied by hypotension (41/20 mmHg) and ST depression in limb lead electrocardiogram (ECG). Atropine 0.5 mg was injected intravenously without improving the hemodynamics. Intravenous adrenaline 0.5 mg was added despite the lack of signs suggesting allergic reactions. Her heart rate and blood pressure quickly recovered and remained stable thereafter, although 12-lead ECG taken 1 h later still showed ST depression.

**Conclusions:**

In this case, the significant bradycardia appeared attributable to coronary vasospasm (Kounis syndrome) induced by sugammadex, considering the ECG findings and high incidence of anaphylaxis due to sugammadex.

## Background

Sugammadex is a cyclodextrin compound designed to reverse the effects of aminosteroidal neuromuscular blocking agents, especially rocuronium [1]; rocuronium is encapsulated in the central core of sugammadex, irreversibly fixed, and neutralized. An acetylcholinesterase inhibitor (e.g., neostigmine) is also used to reverse partial neuromuscular blockade by non-depolarizing muscle relaxants, while acetylcholinesterase inhibition can induce cholinergic effects, including bradycardia. Sugammadex has a safer profile than acetylcholinesterase inhibitors as sugammadex does not cause cholinergic effects [2]. However, the incidence of sugammadex-induced anaphylaxis is relatively high [3]. In addition, several case reports have described profound bradycardia, even cardiac arrest, possibly caused by sugammadex administration, although the mechanism of this rare adverse event has remained unclear [4–9].

Here, we describe a case of severe atropine-resistant bradycardia that occurred after intravenous injection of sugammadex and present a possible cause for this occurrence.

## Case presentation

**Fig. 1:**
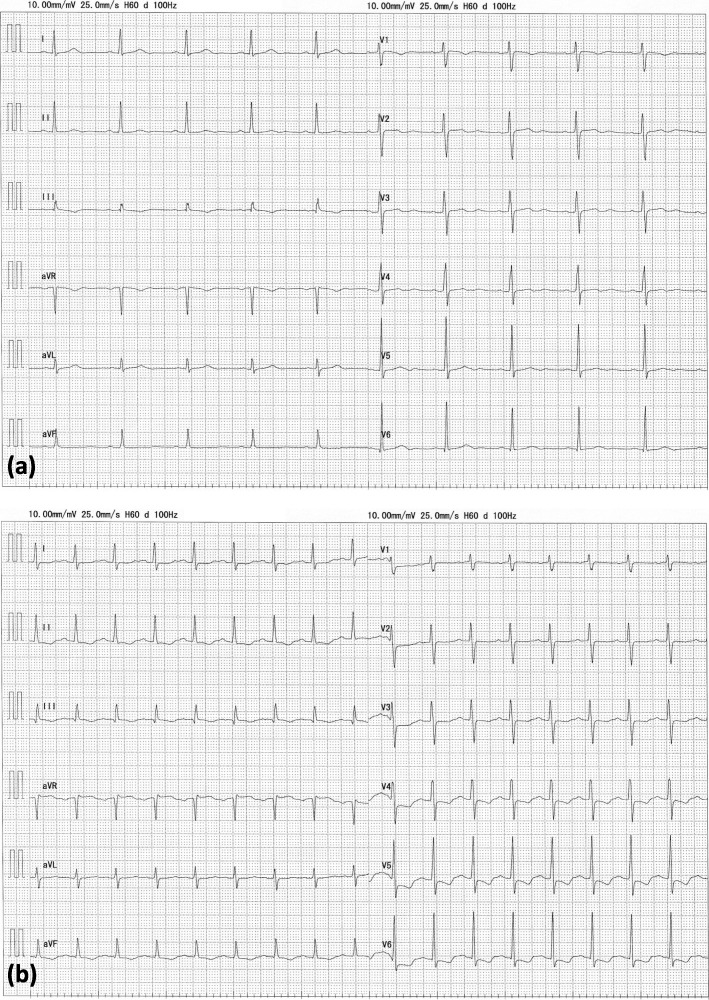
Twelve-lead ECG (electrocardiogram) taken before surgery and at arrival at the intensive care unit (ICU). The preoperative ECG (**a**) showed sinus rhythm (heart rate 62 bpm) with no abnormality. The ECG at arrival at ICU (**b**) showed sinus rhythm (heart rate 102 bpm), whereas it revealed downsloping ST depression in leads II, III, aVF, and V3-6, as well as, ST elevation in lead aVR

A 50-year-old woman (height 156 cm, weight 79.2 kg) was diagnosed with uterine myoma and right ovarian tumor and was scheduled for transabdominal hysterectomy and right salpingo-oophorectomy. The preoperative evaluation showed no comorbidity except obesity. Preoperative 12-lead electrocardiogram (ECG) indicated no abnormality (Fig. 1a). Standard monitoring, including limb lead ECG, non-invasive blood pressure monitoring, and pulse oximetry, was applied when the patient entered the operating room. Before the induction of general anesthesia, an epidural catheter was uneventfully inserted through the intervertebral space between the 12th thoracic vertebra and the first lumbar vertebra. General anesthesia was induced with a target-controlled infusion (TCI) of propofol (target concentration 4.5 μg/ml), continuous infusion of remifentanil 0.25 μg/kg/min, and intravenous fentanyl 200 μg. The patient’s trachea was then intubated following the administration of 50 mg of intravenous rocuronium (Rocuronium Bromide Intravenous Solution®; Maruishi Pharmaceutical Co. Ltd, Osaka, Japan). Intraoperative anesthesia was stably maintained with a TCI of propofol (1.5–2 μg/ml) and infusion of remifentanil (0.05–0.15 μg/kg/min), combined with intermittent boluses and continuous infusion of epidural levobupivacaine 0.25% (total 40 ml). The capnometer and bispectral index (BIS) were also monitored during the management of general anesthesia, while neuromuscular monitoring was not used. BIS ranged 30–50 during surgery. A total of 70 mg of rocuronium, other than the abovementioned 50 mg, was administered during the 177-min surgery.

After surgery, propofol and remifentanil infusions were ceased, and sugammadex (Bridion®; MSD, Tokyo, Japan) 200 mg was intravenously administered. Approximately 1 min after the sugammadex administration, the patient’s heart rate started to decrease from 87 bpm, reaching 36 bpm over 3 min, accompanied by hypotension (41/20 mmHg). ST depression in lead II appeared simultaneously, which was confirmed retrospectively by checking the electronic anesthesia chart, although an anesthesiologist who was in charge of the intraoperative management did not notice it in real-time. Airway pressure under positive pressure ventilation was stable. Atropine 0.5 mg was promptly injected intravenously, but her hemodynamics did not improve. Intravenous adrenaline 0.5 mg was added 2 min after the atropine injection despite the lack of signs suggesting allergic reactions, such as skin rash or urticaria. Her heart rate and blood pressure quickly recovered to 130 bpm and 100/54 mmHg, respectively, and remained stable thereafter. However, the tidal volume of spontaneous breathing fluctuated around 250 ml, leading to hypercapnia (end-tidal CO_2_ 58 mmHg) and alveolar hypoventilation (SpO_2_ 93% [FiO_2_ 1.0]). Neuromuscular monitoring was then applied for the first time, and the train-of-four ratio ranged 0.92–1.07. Chest radiography indicated no abnormalities, and bilateral breath sounds were clear. Furthermore, transthoracic echocardiography, performed approximately 30 min after sugammadex administration, indicated the normal systolic function of both ventricles. Nevertheless, the patient had an inadequate level of consciousness, was unable to follow instructions, and BIS showed 70–80. The patient was subsequently transferred to the intensive care unit (ICU) and sedated, with her trachea intubated to apply 10 cmH_2_O of pressure support.

At arrival of ICU (approximately 1 h after the bradycardia occurred), 12-lead ECG was obtained. It showed sinus rhythm (heart rate 102 bpm), whereas downsloping ST depression in leads II, III, aVF, and V3-6, as well as ST elevation in lead aVR, were noted, implying myocardial ischemia in the broad region (Fig. 1b). Serum tryptase and histamine were not assessed because typical allergic reactions were not observed. After a 9-h overnight observation, we confirmed the adequate recovery of tidal volume and alertness, and then her trachea was extubated. The patient was discharged from the hospital on postoperative day 8 without any sequelae.

## Discussion

In this case, approximately 4 min after sugammadex administration, the patient’s heart rate decreased to 36 bpm accompanied by severe hypotension, which was unresponsive to intravenous atropine but recovered with intravenous adrenaline.

Some case reports have described severe bradycardia associated with sugammadex [4–9]. Sugammadex-induced bradycardia is not due to cholinergic effects as it has been noted even in a patient with a denervated, transplanted heart [8]; however, no other mechanism has been postulated. The use of low-dose adrenaline instead of anticholinergic agents to treat sugammadex-induced bradycardia has been recommended, given the lack of known muscarinic effects of sugammadex [10] and several previous case reports indicating an inadequate response to atropine. Otherwise, even cardiac arrest can occur with sugammadex administration [5, 7].

Anaphylaxis associated with sugammadex is deemed to be relatively high. A single center in Japan reported that the incidence of sugammadex-induced anaphylaxis was 0.059% (95% confidence interval, 0.032–0.10%) [3]. The serum tryptase level would help confirm the diagnosis of anaphylaxis, but it was not measured in our case, owing to a lack of signs of typical allergic reactions. However, technically, anaphylaxis can be diagnosed by just observing severe hemodynamic instability that occurs following the administration of a certain medication, without accompanying tachycardia or skin symptoms, according to the diagnostic criteria for anaphylaxis [11]. In addition, the limb lead ECG monitored in the operating room, and the 12-lead ECG obtained at ICU arrival implied the existence of myocardial ischemia. Hence, we assume that, in the present case, sugammadex administration induced anaphylactic shock with coronary vasospasm, which is known as Kounis syndrome (“allergic angina”) [12–14].

Kounis syndrome is defined as an acute coronary syndrome occurring in association with mast cell degranulation induced by allergic or hypersensitivity insult. It is caused by inflammatory mediators, such as histamine and various cytokines and chemokines released through mast cell activation [14]. Various conditions (e.g., bronchial asthma, mastocytosis), drugs (e.g., antibiotics, analgesics, contrast media, corticosteroids), and environmental exposures (e.g*.*, insects stings, latex contact) could induce Kounis syndrome [12]. Several cases of Kounis syndrome caused by midazolam [15], morphine [16], and rocuronium [17] have been reported. One article described Kounis syndrome probably caused by the rocuronium-sugammadex complex, although this case did not present bradycardia but tachycardia [18]. No published case report regarding sugammadex-induced bradycardia has mentioned Kounis syndrome as a possible cause of bradycardia. The present case showed no typical allergic reactions on the skin or airway. However, according to previous articles, Kounis syndrome occasionally lacks skin signs suggesting allergic reactions [13, 19]. Transthoracic echocardiography in the present case, which indicated normal systolic function, was performed approximately 30 min after recovery from serious hemodynamic instability. Thus, echocardiography finding does not exclude a diagnosis of Kounis syndrome. A previous case report describing profound bradycardia with severe hypotension after sugammadex administration also mentions the simultaneous appearance of ST change on ECG [9]. This report confirmed coronary vasospasm provoked by ergonovine on coronary angiography postoperatively. Saito et al. [20] reported transient third-degree atrioventricular block following sugammadex injection. Therefore, we believe that a considerable proportion of sugammadex-induced bradycardia should involve the mechanism of allergic angina.

The culprit for type 2 respiratory failure seen immediately after recovery from severe bradycardia in the present case remains unclear. The train-of-four ratio demonstrated no residual effect of muscle relaxant, while both chest radiography and lung auscultation showed no abnormality. Therefore, the occurrence of rocuronium recurarization and lung edema induced by anaphylaxis or acute heart failure was unlikely. We speculate that her obesity and concomitant delayed emergence from general anesthesia would have led to the alveolar hypoventilation. Moreover, Kounis syndrome can cause vasospasm not only of the coronary artery but also of the arteries in the brain [21, 22], possibly leading to impaired consciousness as well as central hypoventilation.

We suggest a few countermeasures that should have been taken in this patient to clarify the cause and reduce the risk of bradycardia. First, we should have measured tryptase levels, regardless of the existence of skin symptoms, to distinguish whether the bradycardia was an allergy-related phenomenon or not. Second, 12-lead ECG should have been taken soon after the occurrence of bradycardia to obtain a more precise diagnosis. Furthermore, this patient required additional assessment to confirm the culprit and avoid the occurrence of the same reaction in case of future surgery. This evaluation should have involved coronary angiography including an ergonovine or an acetylcholine provocation test in addition to skin testing to sugammadex.

In conclusion, we believe that significant bradycardia immediately after sugammadex administration was attributable to Kounis syndrome induced by sugammadex based on the time course of the event, ECG findings, and known high incidence of anaphylaxis due to this medication.

## Data Availability

Not applicable due to patient privacy concerns.

## Abbreviations

BIS: Bispectral index
ECG: Electrocardiogram
ICU: Intensive care unit
TCI: Target-controlled infusion
